# An operative case of hepatic pseudolymphoma difficult to differentiate from primary hepatic marginal zone B-cell lymphoma of mucosa-associated lymphoid tissue

**DOI:** 10.1186/1477-7819-9-3

**Published:** 2011-01-13

**Authors:** Michihiro Hayashi, Noboru Yonetani, Fumitoshi Hirokawa, Mitsuhiro Asakuma, Katsuhiko Miyaji, Atsushi Takeshita, Kazuhiro Yamamoto, Hironori Haga, Takayuki Takubo, Nobuhiko Tanigawa

**Affiliations:** 1 Department of General and Gastroenterological Surgery, Osaka Medical College Hospital, 2-7 Daigaku-machi, Takatsuki, Osaka 569-8686, Japan; 2 Division of Comprehensive Medicine, Department of Clinical and Laboratory, Osaka Medical College Hospital, 2-7 Daigaku-machi, Takatsuki, Osaka 569-8686, Japan; 3 Department of Internal Medicine, Osaka Medical College Hospital, 2-7 Daigaku-machi, Takatsuki, Osaka 569-8686, Japan; 4 Department of Pathology, Osaka Medical College Hospital, 2-7 Daigaku-machi, Takatsuki, Osaka 569-8686, Japan; 5 Department of Radiology, Osaka Medical College Hospital, 2-7 Daigaku-machi, Takatsuki, Osaka 569-8686, Japan; 6 Department of Surgical Pathology, Hokkaido University Hospital, North 15, West 7, Kita-ku, Sapporo 060-8638, Japan

## Abstract

Hepatic pseudolymphoma (HPL) and primary hepatic marginal zone B cell lymphoma of mucosa-associated lymphoid tissue (MALT lymphoma) are rare diseases and the differential diagnosis between these two entities is sometimes difficult. We herein report a 56-year-old Japanese woman who was pointed out to have a space occupying lesion in the left lateral segment of the liver. Hepatitis viral-associated antigen/antibody was negative and liver function tests including lactic dehydrogenase, peripheral blood count, tumor markers and soluble interleukin-2 receptor were all within normal limit. Imaging study using computed tomography and magnetic resonance imaging were not typical for hepatocellular carcinoma, cholangiocarcinoma, or other metastatic cancer. Fluorodeoxyglucose-positron emission tomography examination integrated with computed tomography scanning showed high standardized uptake value in the solitary lesion in the liver. Under a diagnosis of primary liver neoplasm, laparoscopic-assisted lateral segmentectomy was performed. Liver tumor of maximal 1.0 cm in diameter was consisted of aggregation of lymphocytes of predominantly B-cell, containing multiple lymphocyte follicles positive for CD10 and bcl-2, consistent with a diagnosis of HPL rather than MALT lymphoma, although a definitive differentiation was pending. The background liver showed non-alcoholic fatty liver disease/early non-alcoholic steatohepatitis. The patient is currently doing well with no sign of relapse 13 months after the surgery. Since the accurate diagnosis is difficult, laparoscopic approach would provide a reasonable procedure of diagnostic and therapeutic advantage with minimal invasiveness for patients. Considering that the real nature of this entity remains unclear, vigilant follow-up of patient is essential.

## Background

A primary hepatic lymphoma (PHL) is defined as lymphoma localized and limited in the liver [1], not the secondary involvement of high- or intermediate grade non-Hodgkin's lymphoma, and accounts for less than 1% of all extranodal lymphomas [2]. Among them, a primary hepatic low-grade marginal zone B cell lymphoma of mucosa-associated lymphoid tissue (MALT lymphoma) is extremely rare.

On the other hand, hepatic pseudolymphoma (HPL), also termed as reactive lymphoid hyperplasia, or nodular lymphoid lesion, is extremely rare disease and characterized by the proliferation of non-neoplastic, polyclonal lymphocytes forming follicles with an active germinal center [3], and most importantly, is mimicking clinicopathologically to low grade lymphoma including MALT lymphoma.

The etiology, pathogenesis and clinical implications of these two diseases remain unknown to a large extent. Reported underlying liver diseases include chronic viral hepatitis, autoimmune liver diseases, etc [4].

Since clinical diagnosis is often difficult especially at its earlier stage, surgical resection appears a mainstay for diagnostic/therapeutic purpose.

We herein present a laparoscopically operated case of hepatic pseudolymphoma which was difficult to differentially diagnose from primary hepatic MALT lymphoma, and discuss the clinicopathological features and clinical implications of these two disease entity.

## Case presentation

In April 2009, a 56-year-old Japanese woman was pointed out to have a space occupying lesion in the lateral segment of the liver on abdominal ultrasonography during health examination. Her social and family history was noncontributory and she had a previous medical history of appendectomy for acute appendicitis and laparoscopic cholecystectomy for cholecystolithiasis. She showed no abnormal physical findings, including lymphadenopathy and hepatosplenomegaly.

Laboratory findings of blood examination were almost normal, including blood cell counts and differentiation, serochemical tests including liver enzymes and lactate dehydrogenase (LDH), hepatitis viral associated markers including hepatitis B virus surface antigen (HBsAg), hepatitis B virus core antibody (HBcAb) and hepatitis C virus (HCV) antibody. Also, tumor markers including carcinoembryonic antigen and carbohydrate antigen 19-9, alpha-fetoprotein (AFP), fucosylated AFP (L3-AFP), protein induced by vitamin-K absence or antagonist II (des-gamma carboxy prothrombin, PIVKA-II), and soluble interleukin 2 (s-IL2) receptor were within normal limits.

Abdominal ultrasonography showed 15-mm-diameter hypoechoic in segment 3 in the liver, and on enhancement study, it showed slight enhancement of ring-like in the peripheral but not in the entire tumor, the center of which being minimally enhanced, which indicated metastatic tumor rather than hepatocellular carcinoma (HCC).

**Figure 1:**
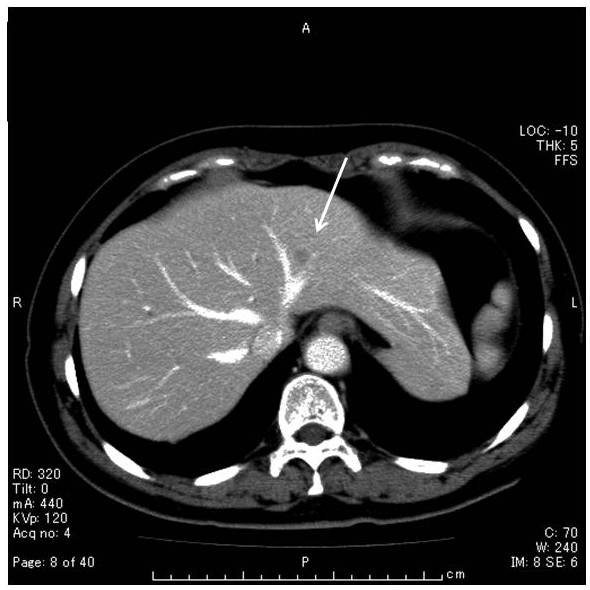
**Unenhanced CT scan showed low density area of 1 cm in diameter in the segment 3 of the liver (arrow)**. Contrast-enhanced CT scan during arterial phase showed minimally peripheral ring enhancement. No lymphadenopathy or hepatosplenomegaly was observed.

On abdominal computed tomography (CT) scan (Figure 1), 15-mm-diameter low density area was demonstrated before contrast material injection, which was enhanced in early arterial phase and subsequently washed out in the late phase after contrast material injection, not incompatible with HCC. Other organs including regional or para-aortic lymph nodes showed no abnormal finding.

**Figure 2:**
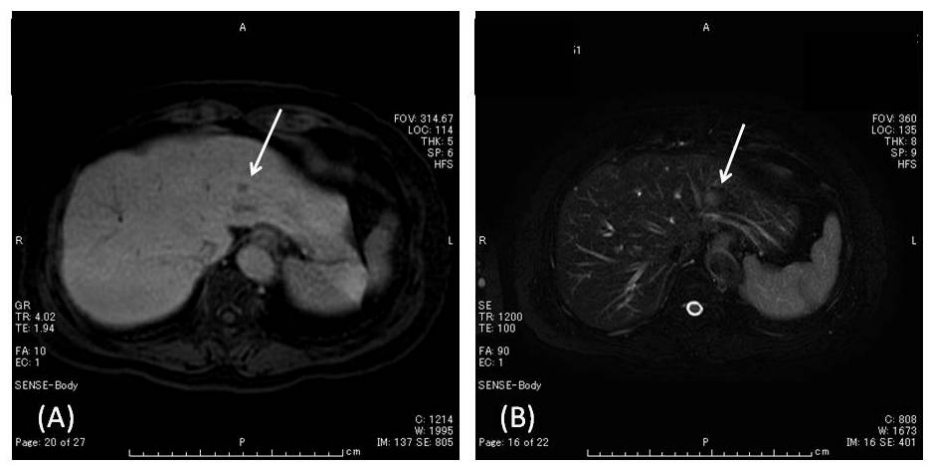
**Magnetic resonance imaging (MRI), the hepatic tumor (arrow) was low signal intensity in T1-weighted image (A) and slight high signal intensity in T2-weighted image (B), and low signal intensity in hepatobiliary phase after Gd-EOB-DTPA injection, and on dynamic Gd-EOB-DTPA MRI protocol not clearly visualized during arterial dominant phase with slight ring-like enhancement persisting, indicating hypovascular tumor, such as cholangiocarcinoma or liver metastasis**.

On magnetic resonance imaging (MRI, Figure 2), the hepatic tumor was low signal intensity in T1-weighted imaging and slight high signal intensity in T2-weighted imaging, and low intensity in hepatobiliary phase 20 minutes after injection of gadolinium ethoxybenzyl diethylenetriamine pentaacetic acid (Gd-EOB-DTPA, Primovist, Bayer Schering Pharma), and on dynamic Gd-EOB-DTPA MRI protocol not clearly visualized during arterial dominant phase and slight ring-like enhancement persisted, indicating hypovascular tumor, such as cholangiocarcinoma or liver metastasis.

On gastric fiberscope examination, atrophic gastritis was noted without evidence of MALT lymphoma. She had previously received eradication treatment for Helicobacter pylori. Colonoscopy examination found no other lesions.

**Figure 3:**
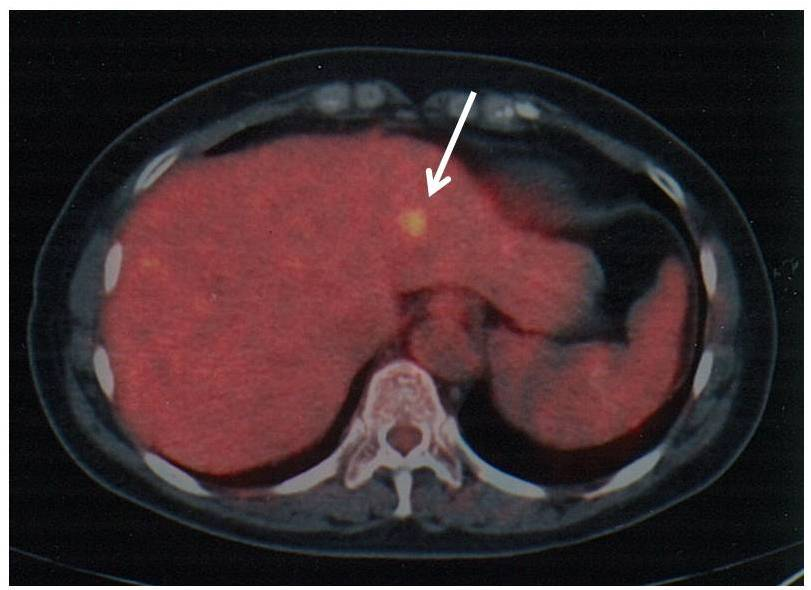
**FDG-PET CT demonstrated the tumor had a high standardized uptake value (SUVmax: 3.6) for FDG**. No other site showed FDG uptake, suggesting that liver tumor is not secondary from malignant lesions of other organs.

In a fluorodeoxyglucose-positron emission tomography examination integrated with computed tomography scanning (FDG-PET CT, Figure 3), the tumor was revealed to have a high standardized uptake value (SUVmax: 3.6) for FDG. No other site showed FDG uptake, suggesting that liver tumor is not secondary from malignant lesions of other organs.

Abdominal angiography revealed small tumor stain in the tributaries of A2 during arterial phase; hence transcatheter arterial infusion of epirubicin and lipiodol was performed under the diagnosis of small HCC.

Percutaneous needle biopsy was performed, but failed to provide with definitive diagnosis regarding the tumor partly due to inappropriate material obtained because of the size of the targeted tumor, except for chronic hepatitis of minimal grade activity and fibrosis (A1/F1, according to the New Inuyama Classification [5], and hepatocyte ballooning with fatty degeneration being noted.

In November 2009, under a clinical diagnosis of primary malignant liver tumor, laparoscopic-assisted lateral segmentectomy was performed.

**Figure 4:**
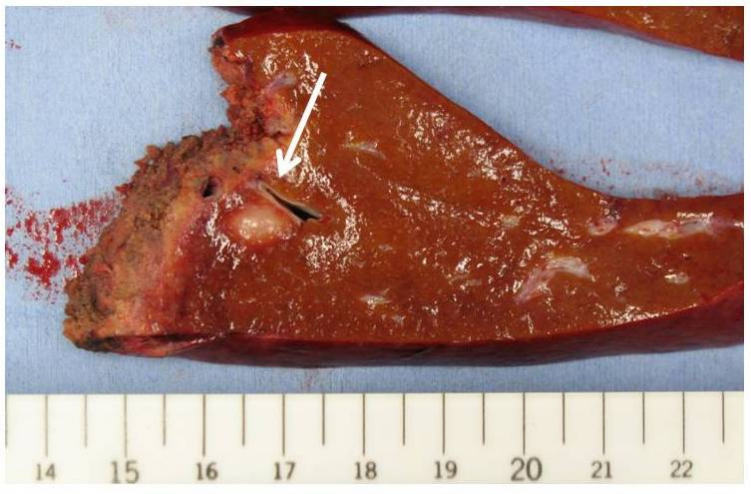
**Resected specimen: section of the liver and 12 × 10 mm, well-defined, light-tan, firm, solid nodule (arrow)**.

**Figure 5:**
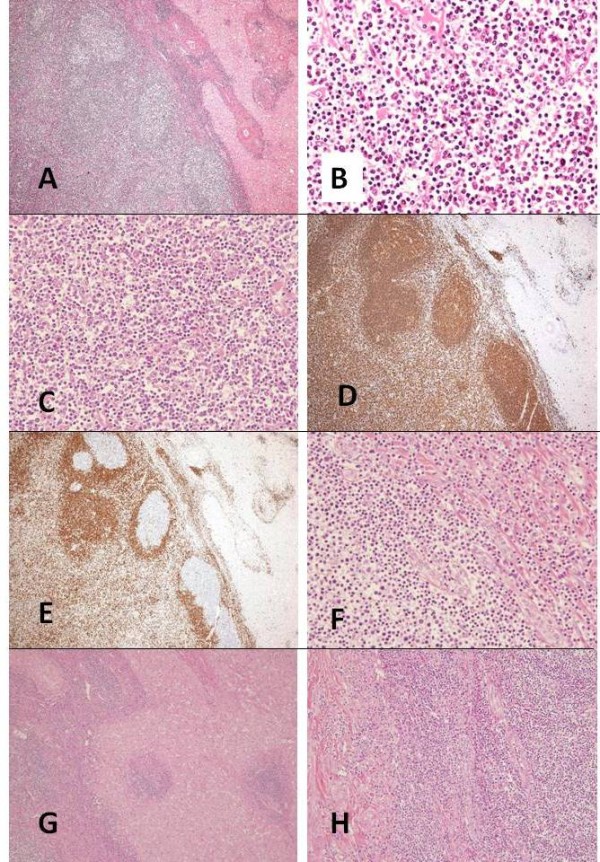
**Microscopic appearance of the lesion**. (A) Low-power view of the tumor showing dense lymphocytic infiltration with lymph follicles (hematoxylin and eosin, original magnification ×4). (B) Interfollicular areas are infiltrated with small to intermediate-sized lymphocytes with pale cytoplasm, which are characteristic features of hepatic pseudolymphoma as well as marginal zone B-cell lymphoma (hematoxylin and eosin, original magnification ×40). (C) Intermediate-sized atypical lymphocytes are observed (hematoxylin and eosin, original magnification ×40). (D) Most of the lymphoid cells are positive for CD20. These cells are negative for CD5, which rules out other small B-cell non Hodgkin lymphomas, including lymphocytic lymphoma and mantle cell lymphoma (original magnification ×4). (E) Bcl-2 expression in the mantle zones and the interfollicular areas (original magnification ×4). (F) Lymphoepithelial lesions are observed at the bile ducts (hematoxylin and eosin, original magnification ×40). (G) At the edge of the nodule, lymphocytic infiltration extends into perinodular portal tracts (hematoxylin and eosin, original magnification ×10). (H) Bile ducts are observed at the periphery of the nodule (hematoxylin and eosin, original magnification ×10).

On macroscopic examination, there was a grey-white solid tumor, measuring 1.0 cm in largest diameter (Figure 4). The tumor was completely excised. On microscopic examination, the tumor in the liver was composed of dense lymphocytic infiltration including multiple lymphoid follicles with germinal centers (Figure 5A). The interfollicular areas were expanded and filled with small to medium-sized lymphocytes with pale cytoplasm and cellular atypia (Figure 5B, C), most of which were positive for CD20 and bcl-2 (Figure 5D, E), and negative for CD5 and CD10. Lymphoepithelial lesions with bile duct epithelium destruction by lymphoid tumor cells were noticed (Figure 5F). At the edge of the nodule, lymphocytic infiltration extended into perinodular portal tracts (Figure 5G). Bile ducts were observed at the periphery of the nodule (Figure 5H). Ki-67 index of those lymphoid cells was 25%. Taken together, these findings were consistent with diagnosis of both extranodal marginal zone B-cell lymphoma and non-neoplastic lesion mimicking MALT lymphoma including HPL, reactive lymphoid hyperplasia, or nodular lymphoid lesion, but still insufficient to distinguish these conditions. Polymerase chain reaction was performed to detect monoclonal immunoglobulin heavy chain (IGH) gene rearrangement in an attempt to differentiate these two entities, but was unsuccessful due to degradation of extracted DNA from the specimen, presumably partly because of deleterious effect on DNA integrity by the previous local tumor ablation using transcatheter arterial chemo-embolization. The background liver showed 30% macrovesicular steatosis with scattered ballooned cells and perivenular fibrosis, suggesting non-alcoholic fatty liver disease (NAFLD) or early non-alcoholic steatohepatitis (NASH). No histological finding suggested concomitant primary biliary cirrhosis (PBC) or viral hepatitis.

The postoperative course of this patient was uneventful and she was discharged from the hospital on postoperative day 8. Any adjuvant chemotherapy or radiotherapy was not indicated, considering that the tumor was confined in the liver with no extrahepatic involvement, and she is currently doing well with no sign of relapse 13 months after the surgery.

## Discussion

Hepatic pseudolymphoma (HPL), also known as reactive lymphoid hyperplasia, or nodular lymphoid lesion, is extremely rare disease entity and so far 35 cases have been reported worldwide [4,6-9] since the first report by Sharifi et al [10]. Its pathogenesis and clinical implications have not been fully elucidated. According to the recently proposed criteria by Zen et al, the present case reported here would be diagnosed with HPL, although not confirmed by molecular examination, such as detection of gene rearrangement.

Primary hepatic marginal zone B-cell lymphoma of the mucosa-associated lymphoid tissue type (MALT lymphoma), also rare entity with only 48 cases being reported in the worldwide literature [11-22] since the first report by Isaacson et al [23], accounts for 1.6-3% of PHL [24,25], is the most important differential diagnosis from HPL.

The etiopathogenesis of HPL remains unclear, although 27% of the patients had chronic liver diseases including HBV- or HCV-related liver cirrhosis, and moreover, 23% had autoimmune disorders, such as PBC, in extrahepatic organs [26]. In terms of background disorders, this figure is comparable with that of MALT lymphoma, where half of the patients have chronic inflammatory liver diseases including autoimmune disorders [11,21,27,28]. The similarity would imply the common pathogenesis of these two conditions. In this regard, several authors have proposed that chronic persistent, prolonged immunogenic stimulation targeted either to infectious agents, such as Helicobacter pylori and HCV, and autoimmune diseases, such as PBC, Hashimoto thyroiditis, and Sjögren syndrome, would induce development of acquired MALT and subsequently MALT lymphoma [28], and/or HPL during this process as well [29].

As a background liver condition associating with HPL, in accordance with the case reported by Zen et al [26], our case would suggest the possible role for NAFLD/NASH as a pathogenesis in this entity through chronic persistent inflammatory stimulation in the liver, though via non-immunological pathway, yet further study is required on this matter.

Clinical resemblance includes the age of patients (mean age, 55.1 vs. 61.4 years old, HPL vs. MALT lymphoma), and tumor characteristics including the size (size range, 0.5-5.5 vs. 2-7.7 cm, HPL vs. MALT lymphoma) and the number, where the majority of cases had solitary tumor at presentation in both entities (81% vs. 78%, HPL vs. MALT lymphoma). The single pronounced difference would be the female preponderance in HPL (86% vs. 51%, HPL vs. MALT lymphoma, 4, 16, 26).

The diagnosis of HPL, not to mention the preoperative one, appears challenging. In fact, the case report by Sato et al indicating the transformation of HPL into lymphoma [30] would implicate the significance, as well as difficulty, of differential diagnosis of these two conditions. In our case, since the lack of molecular diagnosis hampered the definitive diagnosis, there still remains insufficiency in differential diagnosis from hepatic MALT lymphoma [26]. Therefore, molecular analysis should be routinely undertaken as the most potent diagnostic tool in such a controversial case. However, at the same time, it should be noted that even a clonal IGH rearrangement would not be a gold standard for a diagnosis of lymphoma, as suggested by Geyer et al. in the setting of lower female genital tract, and therefore both routine microscopic findings and detailed clinical information remain paramount in establishing the correct diagnosis [31]. In this context, the clinical course of this patient on careful follow-up hereafter might elucidate the essentials of this disorder.

Regarding the diagnostic significance of FDG-PET, no report on HPL is currently available. In contrast, FDG-PET has been reported in the recent two reports as a useful and convenient modality for the diagnosis of hepatic MALT lymphoma [21,22]. Considering the positive uptake of FDG seen in our case, however, this examination would not be used solely to differentiate the two conditions.

Natural history of HPL is yet delineated. Malignant transformation of pseudolymphoma into true lymphoma has been reported in the various organs, such as lung, stomach, and skin [32-34], but actual frequency have not evaluated, since the majority of cases underwent surgical resection under a suspicion for malignancy. Regarding liver, since only one case report is available [30], the possibility of transformation of HPL into hepatic MALT lymphoma could not be determined. Therefore, surgical removal is the treatment of choice for these conditions [35]. Further accumulation of clinical data is required to clarify this matter.

With respect to treatment strategy, reported cases demonstrated that even MALT lymphoma has a favorable prognosis compared with other subtypes of PHL; the former is usually limited to the liver and surgical resection cures the patient in most cases [36]. Also, when diagnosis is confirmed after needle biopsy, non-resection treatment procedures would be permitted, such as radiofrequency ablation [37] instead of surgical resection. Furthermore, in case of HPL, simple observation proved to be practically enough since spontaneous diminution of tumor size or even regression of tumor has been reported [26,38], yet further accumulation of data is needed.

Regarding prognosis after treatment, since no recurrence of MALT lymphoma has been reported to date to occur after adequate surgical resection or chemotherapy treatments [21], even if the tumor is true neoplastic lesion, as long as it remains low-grade malignancy, the surgical outcome would be comparable with that for HPL. However, it should be noted that there is a single report of local recurrence after surgical resection, suggesting the importance of close post-treatment follow-up [39]. Considering the relatively short duration of observation period in the reported cases, vigilant follow-up of the patients including our case would be required.

PHL and lymphoid lesions in general should be considered in the differential diagnosis of space occupying lesions of the liver in the absence of elevated levels of ordinary tumor markers including AFP and CEA [40]. In addition, it is also important to distinguish HPL and MALT lymphoma from others, particularly from more aggressive type, such as mantle cell lymphoma [41]. However, because of their indolent, localized clinical presentation, diagnosis is often accompanied by substantial difficulty, with the majority of cases being diagnosed incidentally. Admitted that even in case of lymphoma, there is a certain chance of cure by means of medical treatment without surgery, en bloc resection of the hepatic tumor would be recommended as a principle procedure for subsequent diagnosis and decision for treatment. Needle biopsy failed to present a diagnosis in our case, partly because the tumor was among the smallest of the reported cases in the literatures. Finally, in such situation, laparoscopic approach would provide a reasonable procedure of less invasiveness for patients [42].

## Conclusion

HPL and MALT lymphoma are very rare. We herein report a case with a space occupying lesion in the left lateral segment of the liver, which was completely resected by laparoscopic-assisted lateral segmentectomy. The tumor of maximal 1.0 cm in diameter was consisted of aggregation of lymphocytes of predominantly B-cell, containing multiple lymphocyte follicles positive for CD10 and bcl-2, consistent with a diagnosis of HPL, but still necessitating differential diagnosis from MALT lymphoma. Since the accurate diagnosis of this entity is difficult, laparoscopic approach would provide a reasonable procedure of diagnostic and therapeutic advantage with minimal invasiveness for patients. Considering that the real nature of this entity remains unclear to date, vigilant follow-up of patient is essential.

## Consent

Written informed consent was obtained from the patient for publication of this case report and any accompanying images. A copy of the written consent is available for review by the Editor-in-Chief of this journal.

## List of abbreviations

HPL: hepatic pseudolymphoma; MALT: mucosa-associated lymphoid tissue; PHL: primary hepatic lymphoma; LDH: lactate dehydrogenase; s-IL2: soluble interleukin 2; CEA: carcinoembryonic antigen; AFP: alpha-fetoprotein; HCV: hepatitis C virus; CT: computed tomography; MRI: magnetic resonance imaging; HCC: hepatocellular carcinoma; Gd-EOB-DTPA: gadolinium ethoxybenzyl diethylenetriamine pentaacetic acid; FDG-PET: fluorodeoxyglucose-positron emission tomography; SUV: standardized uptake value; IGH: immunoglobulin heavy chain; NAFLD: non-alcoholic fatty liver disease; NASH: non-alcoholic steatohepatitis; PBC: primary biliary cirrhosis.

## Competing interests

The authors declare that they have no competing interests.

## Authors' contributions

MH conceived the study concept and design, was involved with patient care and drafted the manuscript and literature review. NY, FH, MA, KM, AT, KY HH, and TT were involved with formation of the study concept and design, patient care and drafting of the manuscript and literature review. NT carried out the operation on the patient and was the main contributor in the writing of the manuscript. All authors have read and approved the final version of the manuscript.
